# First Evidence of Functional Neuronal Remodeling In Vitro in a Cell Line from an Evolutionarily Ancient Vertebrate (Sturgeon)

**DOI:** 10.1007/s10571-026-01783-x

**Published:** 2026-07-22

**Authors:** Bianka Grunow, Jonna Schulz-Ehlbeck

**Affiliations:** Workgroup Fish Growth Physiology, Research Institute for Farm Animal Biology (FBN), Wilhelm-Stahl-Allee 2, 18196 Dummerstorf, Germany

**Keywords:** AOXlar7y, Neuronal differentiation, Fish cell line, NGF, Calcium activity, ATP stimulation

## Abstract

The targeted differentiation of stem cell-like cells into functional neurons is a central goal in developmental biology and comparative neurobiology. In this study, we report for the first time on the successful neuronal remodeling of cell line derived from the Atlantic sturgeon larvae (*Acipenser oxyrinchus;* AOXlar7y) under defined in vitro conditions. The cells were cultured on poly-D-lysine-coated surfaces and exposed to a reduced-serum medium supplemented with nerve growth factor-β to induce neuronal differentiation. Morphologically, time-dependent formation of neurite-like processes and network structures was observed over a period of 14 days. The neuronal identity of the differentiated cells was confirmed by immunocytochemistry with the neuronal marker NeuN. Functional maturation was examined by measurement of cytoplasmatic calcium activity. After stimulation with 200 µM ATP, cells with neuronal morphology showed distinct intracellular calcium transients, indicating functional purinergic signaling pathways. AOXlar7y thus represents a novel in vitro model for studying neurogenesis and neuronal function in a cellular model of ancient fish species and provides a valuable basis for future studies in comparative neurobiology, neurotoxicology, and evolutionary developmental biology.

**Graphical Abstract:**

First successful remodeling of a fish cell line into neural cells. Morphological changes, functional activity as demonstrated by calcium recordings, and the expression of neural markers by immunofluorescence confirm the successful neural-like remodeling and functional maturation of the Atlantic sturgeon cell line.
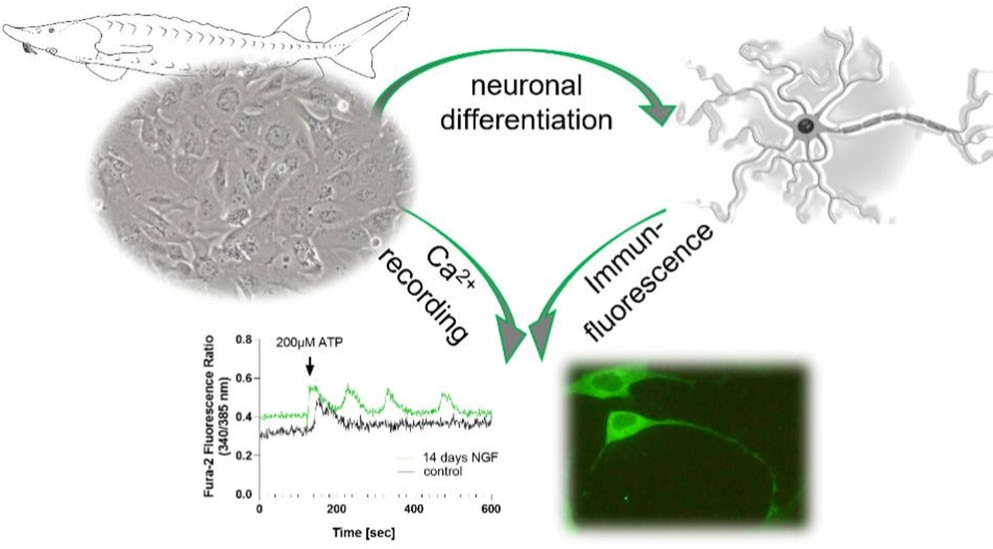

**Supplementary Information:**

The online version contains supplementary material available at 10.1007/s10571-026-01783-x.

## Introduction

The differentiation of stem cells into neurons is a fundamental process in vertebrate development and a key area of interest in regenerative biology, medicine, and pharmacology (Park et al. 2023). Although much of our current knowledge is derived from traditional model organisms such as mice and zebrafish (Kalueff and Stewart et al. 2014; Azkona and Sanchez-Pernaute 2022), the use of alternative species can provide valuable evolutionary and comparative insights (Ganz and Brand 2016). In this context, non-model fish species are increasingly recognized as important systems for studying neurogenesis under diverse physiological and environmental contexts (Gomez-Mercader et al. 2024). Notably, the formation of functional neurons from neural stem cells in the adult central nervous system is widespread among vertebrates and especially prominent in non-model fish. This offers valuable opportunities for studying neuronal differentiation and regeneration processes (Zupanc 2021).

The Atlantic sturgeon (*Acipenser oxyrinchus*), one of the oldest vertebrates, offers unique insights into the biology of early-branched fish. The establishment of stable cell lines of this species has opened up new avenues for in vitro research (Yebra-Pimentel et al. 2019; Grunow et al. 2021; Lutze et al. 2024; Di Leonardo et al. 2025). The AOXlar7y cell line, has therefore emerged as a promising tool for studying cellular plasticity and differentiation potential in a non-model fish context.

Differentiation of stem cells into physiologically active neurons represents an important milestone in developmental and regenerative biology. While differentiation protocols for mammalian cell lines are already well established (Martorana et al. 2018), there are no comparable protocols for fish cell lines. This limits both comparative developmental studies and research into fish-specific neurobiological processes in vitro.

Here, we report for the first time the successful conversion of stem cells into physiologically active nerve cells, accompanied by neuronal-like morphological remodeling, in a fish cell culture system. Using the AOXlar7y cell line derived from Atlantic sturgeon, we have established a protocol that induces differentiation into neuronal cells. Neuronal identity was confirmed by morphological studies and by the detection of electrophysiological activity. This breakthrough not only establishes the AOXlar7y cell line as a novel in vitro model for fish neurogenesis, but also highlights the broader potential of non-model aquatic species in neurodevelopmental research. By expanding the available cellular systems beyond traditional vertebrate models, our study opens new avenues for evolutionary, toxicological, and translational neuroscience.

## Methods

### Cell Culture and Maintenance

The AOXlar7y is a spontaneously immortalized cell line derived from Atlantic sturgeon whole larvae (*Acipenser oxyrinchus;* CVCL_WC97, Bairoch (2018). This cell line, established and obtained from the German Cell Bank for Wildlife (Fraunhofer EMB, Lübeck, Germany; Grunow et al. 2011), was cultured under optimal standard conditions at 20 °C in Leibovitz’s-15 medium (Gibco, Germany) supplemented with 10% fetal bovine serum (FBS, PAN-Biotech, Germany) and 1% penicillin-streptomycin (P/S; Gibco Life Technologies, Germany), (Lutze et al. 2024). Cells in passage 32 to 47 and 60 to 80 were used. Cells were passaged at 70–80% confluence.

### Experimental Setup for Neuronal Cell Formation

To identify optimal conditions for neuronal differentiation, we systematically varied four key parameters: surface coating, seeding density, serum concentration, and nerve growth factor- β (NGF-β) supplementation.

Cells were seeded on uncoated tissue culture plastic (TPP, Techno Plastic Products AG, Switzerland), 0.1% gelatine-coated (Serva, Germany), or poly-D-lysine (0.01%, Merck, Germany) coated TPP-plastic. Coatings were applied according to standard protocols and allowed to dry prior to cell seeding. Cell seeding densities of 15,000, 20,000, 25,000, or 30,000 cells/cm² were chosen to ensure sufficient attachment and cell–cell interaction during the early differentiation phase. To evaluate the impact of serum on differentiation, 0% and 10% FBS concentrations were tested during the 24 h cell attachment phase. During subsequent cultivation, medium was replaced containing 0%, 1%, 2.5%, or 5% FBS. Recombinant human NGF-β from rat (Merck, Germany) was added to the medium at concentrations of 0 ng/ml (control), 100 ng/ml, or 200 ng/ml. These differentiation media were refreshed every 2–3 days. Each experimental approach was tested twice, using 2 × 2 wells per run, resulting in data based on 8 wells in total, including four biological replicates.

### Assessment of Neuronal-like Properties

Neuronal differentiation was evaluated by changes in cell morphology (e.g., neurite-like outgrowth). To analyse the morphology of the AOXlar7y cells, phase contrast microscopic pictures (AE2000, Motic^®^, Barcelona, Spain) were taken after 24 h, after 6 and 14 days for each condition with Moticam 5 Plus in combination with Motic Images Plus 3.0 Software (Motic^®^, Barcelona, Spain). For brightness and contrast, pictures were further processed with Adobe Photoshop CC 2019 (Adobe Inc., California, USA). For all subsequent analyses, only the optimal approach was used: poly-D-lysine coating, seeding at 15,000 cells/cm² with 0% FBS for 24 h, and differentiation in L-15 medium with 1% FBS and 100 ng/ml NGF-β. The ratio of differentiated (neurite out-growth) to undifferentiated cells (epithelial-like morphology) was calculated in six control cultures (725 ± 91.9 cells/image) and twenty cultures of NGF-treated AOXlar7y cells (33.55 ± 10.9 cells/image). The neurite length was only measured if it was at least twice the size of the cell body diameter, with a total of 208 neurites analysed in these 20 images/cultures with the software CellSens Dimension version 3.2 (Evident, Hamburg, Germany).

#### Immunfluorescence

AOXlar7y cells (control – non-differentiation and differentiated cells) were fixed using cold methanol/acetone (3:7) for 10 min and blocked with 2% BSA in PBS for one hour at RT. Primary antibodies against NeuN (rabbit polyclonal, 1:500; Proteintech, catalog 26975-1-AP) were incubated overnight at 4 °C. The antibody against NeuN has previously been successfully tested and validated in Western blot analyses on fish tissue (Supplemental 1). The secondary antibody (Goat Anti‑Rabbit IgG [H + L], conjugated to Alexa Fluor 488, Abcam, catalog ab150077) was applied for one hour at room temperature. Nuclei were counterstained with DAPI (1:100).

### Recording of Cytoplasmic Calcium Dynamics

To assess the physiological activity of differentiated and non-differentiated AOXlar7y cells, intracellular calcium dynamics were measured using ratiometric calcium imaging with the fluorescent dye Fura-2/AM as described by Walter et al. (2015) with a FURA-2 loading at RT (20–22 °C). Only cells that displayed clear neuronal morphology were selected for calcium analysis after 14 days of NGF-β treatment.

During experiments, differentiated and non-differentiated AOXlar7y cells were maintained in standard external solution (140 mM NaCl, 2.7 mM KCl, 1.5 mM CaCl₂, 1 mM MgCl₂, 12 mM HEPES, 6 mM glucose, pH 7.4) or Ca²⁺-free solution (without CaCl₂). Cytosolic Ca²⁺ levels were monitored using Fura-2/AM (2 µM, 45 min at  RT). After washing and 30 min recovery, cells were imaged in Ca²⁺-free solution using an Axiovert 200 microscope (BFi OPTiLAS, Puchheim, Germany). Fluorescence at 340/380 nm excitation and > 510 nm emission was recorded every 10s. At minute two, 200 µM ATP was applied to the cells to stimulate purinergic receptors and induce calcium signalling. The response was monitored for an additional eight minutes interval. A minimum of six independent experiments with at least 2 cells were analysed using two-tailed Mann–Whitney U test (GraphPad Prism 11.0.0.).

## Results and Discussion

**Fig. 1:**
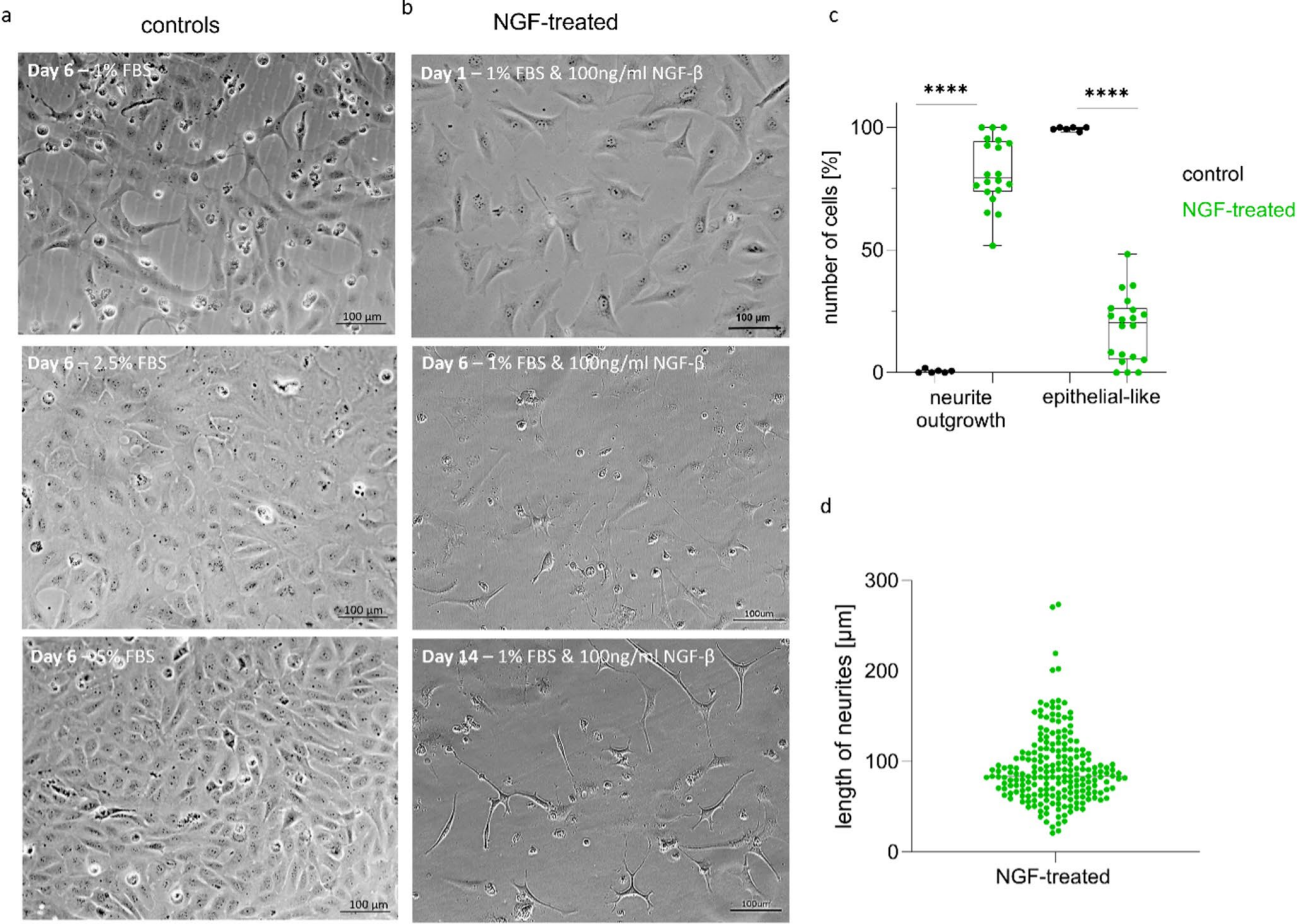
Neuronal formation of AOXlar7y cells under control and NGF treated conditions. The cells were seeded on poly-D-lysine-coated surfaces at a density of 15,000 cells/cm². During the first 24 h, the cells were kept in serum-free (0% FBS) L-15 medium to facilitate cell adhesion. From day 1 onwards, the AOXlar7y cells were kept under the various experimental conditions. **a**-left column: Control cells cultured for 6 days in L-15 medium with 1%, 2.5%, or 5% FBS without NGF-β supplementation. At 1% FBS, the cells did not survive longer than 14 days. In contrast, AOXlar7y cells at 2.5% and 5% FBS showed proliferation but no signs of differentiation-neurite-like outgrowth. **b** – middle column: AOXlar7y cells cultured in L-15 medium with 1% FBS and 100 ng/mL NGF-β, photographed on days 1, 6, and 14. Over time, the cells developed pronounced neurite-like extensions and interconnected networks, indicating progressive neuronal maturation. Scale bar = 100 μm. **c** Percentage of cells with neurite outgrowth compared to epithelial morphology in standard culture (black dots) and under differentiation conditions (green dots). Statistical comparisons were performed using one-way ANOVA, followed by Tukey’s multiple comparison test with **** *p* < 0.0001. **d** Neurite length distribution in NGF-treated cells (mean ± SD: 92.31 μm; range: 21.03–273.05 μm; *n* = 208 neurites)

**Fig. 2:**
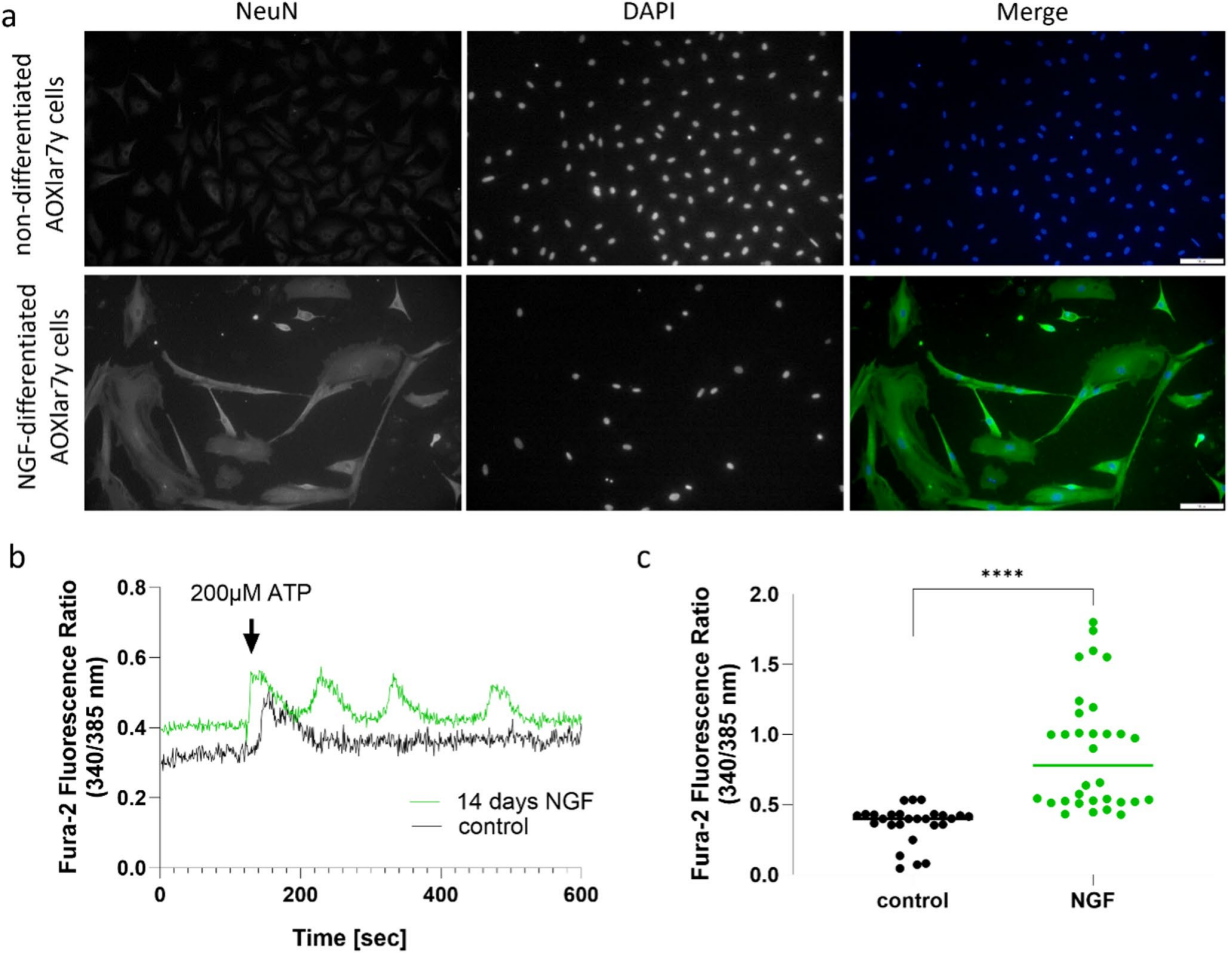
Comparative analysis of NGF-differentiated and control AOXlar7y cells with respect to neuronal marker expression and functional calcium responses. **A** displays immunocytochemical staining for NeuN and DAPI. Upper images show the non-differentiated control cells, and lower images show NGF-β‑treated, differentiated cells. NeuN and DAPI are presented in grayscale for clarity, with merged images in color (NeuN in green; excitation 472 nm, emission 520 nm and nuclei in blue; excitation 377 nm, emission 447 nm) to highlight colocalization. Images were acquired at room temperature (20–22 °C) using a BZ-9000 microscope (Keyence) with a PlanAPO-40x objective, captured with BZ2-Viewer, and further analysed using BZ-Analyzer 2.1. (Keyence). Scale bars: 100 μm. **B** presents representative time courses of the 340:380 ratio in one differentiated (green curve) and one undifferentiated cell (control, black curve), revealing differences in response latency, slope, and amplitude. Cells were first recorded under basal conditions for two minutes, and then stimulated with 200 µM ATP (indicated by an arrow). **C** shows the quantification of peak increases in the 340:380 ratio upon ATP stimulation in differentiated vs. control cells. The resulting changes in intracellular calcium concentration ([Ca²⁺]_i_) were monitored using the fluorescence emission ratio at 340 nm and 380 nm (F₃₄₀/F₃₈₀). As both groups deviated from normal distribution (Shapiro–Wilk test), differences between groups were analyzed using a two-tailed Mann–Whitney U test. A significant increase was observed in the NGF group compared to control (U = 37, *p* < 0.0001). Median values were 0.3995 (*n* = 27) for control and 0.7792 (*n* = 32) for NGF treated cells. The horizontal line indicates the median, and individual data points represent single measurements. The median difference (Hodges–Lehmann estimate) was 0.4692

In this study, we successfully demonstrated the formation of the Atlantic sturgeon-derived AOXlar7y cell line into physiologically active cells with neuronal-like morphology under defined in vitro conditions. AOXlar7y cells changed their morphology under optimized culture conditions involving poly-D-lysine coating, controlled seeding of 15,000 cells/cm^2^ with 0% FBS concentrations during the first 24 h and a differentiation medium containing 1% FBS and 100 ng/ml NGF-β in L-15 basal medium. Phase-contrast microscopy revealed that the epithelial-like morphology changed to cells with neurite-like extensions and network formation (Fig. 1a, b). In control cultures, only 0.56% of cells showed small outgrowths that were too short for definitive measurement; the remaining 99.4% were purely epithelial. (Fig. 1c). In NGF-treated cultures, 81.97% of cells exhibited neurite outgrowths with a mean length of 92.31 μm (range: 21.03–273.05 μm), while 18.03% of cells showed no outgrowths (Fig. 1c, d). Immunofluorescence staining confirmed the expression of the neuronal marker NeuN in differentiated cells, supporting successful neuronal commitment (Fig. 2A). This establishes the AOXlar7y cell line as a novel and valuable in vitro model for studying fish neurogenesis, expanding the repertoire of non-traditional vertebrate models in neurobiological research (Smith et al. 2018; Johnson and Lee 2020).

To evaluate functional maturation, calcium imaging was performed after stimulation with 200 µM ATP. Differentiated AOXlar7y cells showed strong intracellular calcium signals that were significantly higher than those observed in undifferentiated control cells (Fig. 2B–C). ATP is an important signaling molecule in the nervous system. It influences synaptic communication, neuronal activity, and cell function via P2 receptors (Burnstock 2007; Khakh and North 2012). The increase in P2 receptor expression caused by NGF-β treatment likely promotes calcium influx from outside the cell and release from internal stores. This may indicate that the cells have developed a more neuron-like calcium signaling system. These calcium responses suggest that differentiated AOXlar7y cells may not only resemble neurons morphology, but also begin to exhibit neuron-like functional properties. However, further studies are required to substantiate this interpretation.

Our results confirm previous reports highlighting the role of extracellular ATP as a potent modulator of intracellular calcium dynamics in various neuronal cell types (Fields and Burnstock 2006). In fish models, purinergic signaling has been linked to neurological developmental processes, neuroprotection, and responses to injury, but in vitro evidence in fish-derived neuronal cultures remains limited (Li et al. 2021). The present data showed distinct ATP responses between control and differentiated cells. In controls, ATP induced a single transient Ca²⁺ peak, suggesting activation of a purinergic receptor that opens only once, possibly due to rapid desensitization. However, differentiated cells showed oscillating Ca²⁺ signals, suggesting repeated channel openings and implying the expression of a different purinergic receptor or altered signaling dynamics. These data thus represent a fundamental step toward investigating purinergic mechanisms in sturgeon neurons, with potential implications for understanding evolutionary aspects of vertebrate neurophysiology.

Further studies are needed to address current limitations, such as the characterization of specific purinergic receptor subtypes expressed in AOXlar7y-derived neurons and the investigation of their downstream signaling pathways. In addition, electrophysiological recordings and synaptic marker analyses are required to complement the calcium imaging data and provide a comprehensive picture of neuronal functionality. While these approaches will be essential to ultimately demonstrate full neuronal identity in future work, the present study findings already represent a strong step toward this goal.

## Conclusion

In summary, this work demonstrates that stem-like cells from a non-model fish species can be directed towards functional neuronal phenotypes, with ATP-responsive calcium signalling serving as a key functional read-out. This opens new avenues for comparative neurobiology and the study of neuronal differentiation and signalling in evolutionarily distinct vertebrate systems.

## Supplementary Information

Below is the link to the electronic supplementary material.

Supplementary Materila 1. Western blot analysis of NeuN expression. Twenty µg of total protein per lane were loaded. A specific band corresponding to NeuN (~46 kDa) was detected using a rabbit polyclonal anti-NeuN antibody (Proteintech, Cat#26975-1-AP). To assess antibody specificity in fish species, protein extracts were obtained from yolk sac larvae of Acipenser guldenstaetii (AGU), Acipenser baerii (ABA), and a hybrid of both (ABA+AGU). Brain tissue from adult pikeperch (Sander lucioperca, SLU) and adult herring (Clupea harengus, CHA) was included as positive controls.

## Data Availability

All relevant data are provided within the manuscript.

## Abbrevations

ATP: Adenosine triphosphat
AOXlar7y: Cell line from Atlantic sturgeon larvae no.7 (*Acipenser oxyrinchus*)
DAPI: 4′,6-Diamidin-2-phenylindol
FBS: Foetal Bovine Serum
Fura2/AM: Calcium-indicating dye in the acetoxymethyl ester form
L-15: Leibovitz-15
NGF-β: Nerve growth factor -beta
NeuN: Neuronal nuclei (neuron-specific nuclear marker)
P/S: Penicillin/streptomycin
PBS: Dulbecco’s Phosphate-Buffered Saline
TPP: Techno Plastic Products AG
