# Supplementary figures and images for: First Evidence of Functional Neuronal Remodeling In Vitro in a Cell Line from an Evolutionarily Ancient Vertebrate (Sturgeon)
Source: Cell Mol Neurobiol. 2026 Jul 22;46:120. doi: 10.1007/s10571-026-01783-x (PMC13391976; doi:10.1007/s10571-026-01783-x)

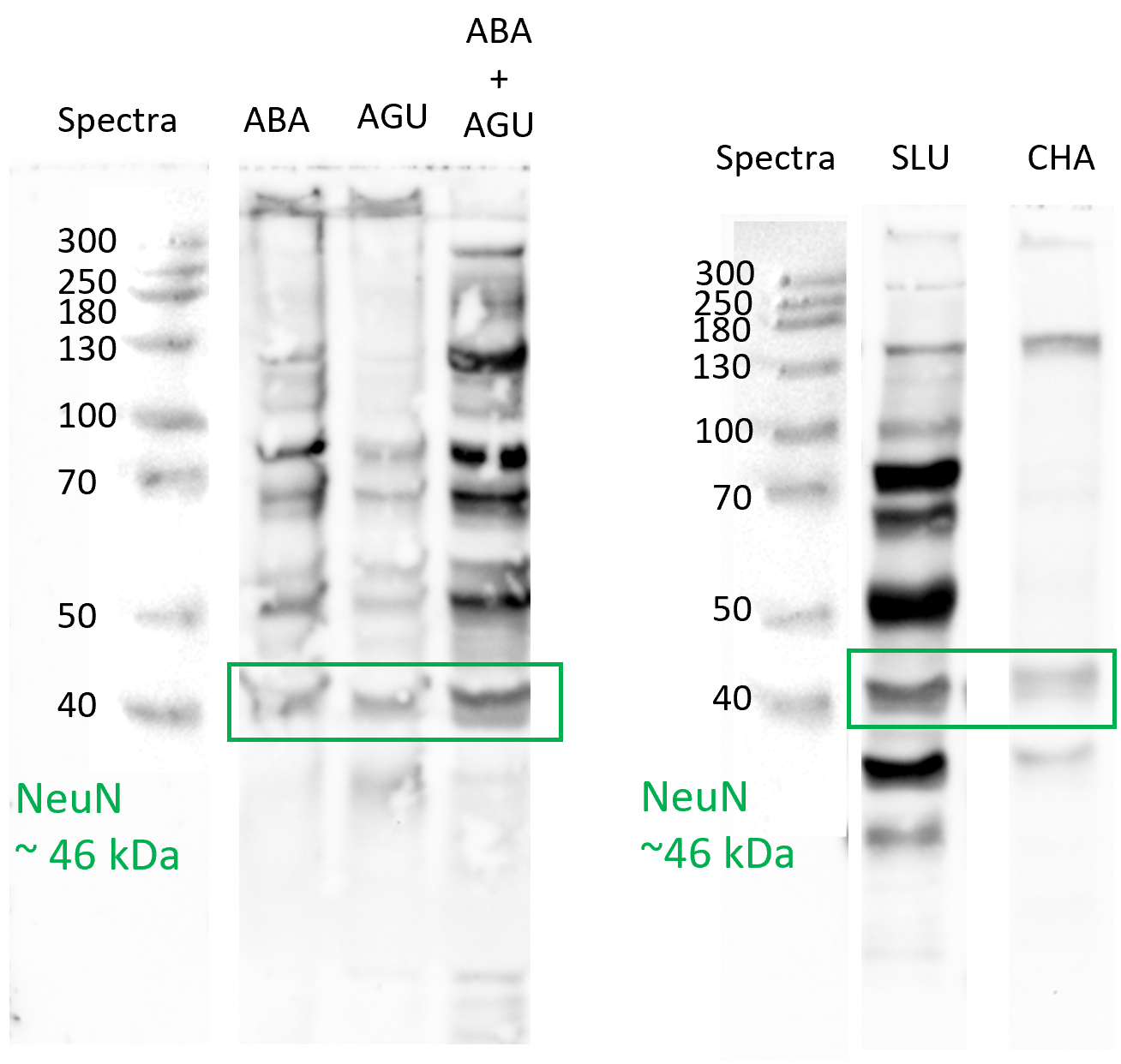

Supplement: Supplementary file 1 — Supplementary Materila 1. Western blot analysis of NeuN expression. Twenty µg of total protein per lane were loaded. A specific band corresponding to NeuN (~46 kDa) was detected using a rabbit polyclonal anti-NeuN antibody (Proteintech, Cat#26975-1-AP). To assess antibody specificity in fish species, protein extracts were obtained from yolk sac larvae of Acipenser guldenstaetii (AGU), Acipenser baerii (ABA), and a hybrid of both (ABA+AGU). Brain tissue from adult pikeperch (Sander lucioperca, SLU) and adult herring (Clupea harengus, CHA) was included as positive controls. [file 10571_2026_1783_MOESM1_ESM.png]
